# The Food and Beverage Occurrence of Furfuryl Alcohol and Myrcene—Two Emerging Potential Human Carcinogens?

**DOI:** 10.3390/toxics5010009

**Published:** 2017-03-11

**Authors:** Alex O. Okaru, Dirk W. Lachenmeier

**Affiliations:** 1 Department of Pharmaceutical Chemistry, University of Nairobi, P.O. Box 19676-00202, Nairobi, Kenya; alex.okaru@gmail.com; 2 Chemisches und Veterinäruntersuchungsamt (CVUA) Karlsruhe, Weissenburger Strasse 3, 76187 Karlsruhe, Germany

**Keywords:** furfuryl alcohol, β-myrcene, carcinogens, occurrence

## Abstract

For decades, compounds present in foods and beverages have been implicated in the etiology of human cancers. The World Health Organization (WHO) International Agency for Research on Cancer (IARC) continues to classify such agents regarding their potential carcinogenicity in humans based on new evidence from animal and human studies. Furfuryl alcohol and β-myrcene are potential human carcinogens due to be evaluated. The major source of furfuryl alcohol in foods is thermal processing and ageing of alcoholic beverages, while β-myrcene occurs naturally as a constituent of the essential oils of plants such as hops, lemongrass, and derived products. This study aimed to summarize the occurrence of furfuryl alcohol and β-myrcene in foods and beverages using literature review data. Additionally, results of furfuryl alcohol occurrence from our own nuclear magnetic resonance (NMR) analysis are included. The highest content of furfuryl alcohol was found in coffee beans (>100 mg/kg) and in some fish products (about 10 mg/kg), while among beverages, wines contained between 1 and 10 mg/L, with 8 mg/L in pineapple juice. The content of β-myrcene was highest in hops. In conclusion, the data about the occurrence of the two agents is currently judged as insufficient for exposure and risk assessment. The results of this study point out the food and beverage groups that may be considered for future monitoring of furfuryl alcohol and β-myrcene.

## 1. Introduction

The production and processing of foods and beverages may invariably lead to significant changes in the chemical composition of the products. The Maillard reaction—which yields furanic compounds such as furfural and 5-hydroxymethylfurfural (HMF) and furfuryl alcohol, among other products—is common during processes that involve heating or roasting [1,2,3,4,5]. Furfuryl alcohol is a food contaminant which occurs in significant amounts in thermally processed foods such as coffee, fruit juices, baked foods; in cask-stored alcoholic beverages such as wines, wine-derived spirits such as brandy, and whiskies as a result of enzymatic or chemical reduction of furfural [6,7,8]; and in butter and butterscotch when furfuryl alcohol is used as a flavouring agent [9]. Furfuryl alcohol may also be formed from quinic acid or 1,2-enediols as precursors during the heating of foods such as coffee beans [5] . In acidic conditions, furfuryl alcohol polymerizes to aliphatic polymers that give a brown colouration to foods [5]. 

Myrcene is a terpenoid compound that exists in two forms—β and α, with the former occurring naturally in essential oils of plants such as hops, bay, lemongrass [10,11], and orange juice [12], and is permitted for use as a flavouring additive of food both by the United States Food and Drug Administration (FDA) since 1965 and by the European Council since 1974. β-Myrcene is also an ingredient in the preparation of olefinic scents such as menthol, and the alcohols linalool, nerol, and geraniol [13], found in household items. 

Analysis of furfuryl alcohol can be done by either gas and liquid chromatography with UV, biosensor, or fluorescence detection [5,6,14,15,16,17], while β-myrcene is typically determined using gas chromatography with mass spectrometry or flame ionization detection [18,19,20,21].

Diet is considered to be the greatest source of human exposure to furfuryl alcohol and β-myrcene. However, unlike the furanic compounds furan, 5-hydroxymethylfurfural (HMF), and furfural, and other food and beverage constituents such as ethanol, ethyl carbamate, or polycyclic aromatic hydrocarbons for which extensive occurrence data is available [22,23,24,25,26], there is a paucity of information on human dietary exposure to furfuryl alcohol and β-myrcene. The two agents are due for assessment as to their carcinogenicity by the International Agency for Research in Cancer (IARC) expert working group in their meeting to be held in June 2017. This study aims to provide an overview of the occurrence of furfuryl alcohol and β-myrcene in foods and beverages.

## 2. Materials and Methods

Occurrence data on furfuryl alcohol and β-myrcene were searched in the following databases: PubMed, Toxnet and ChemIDplus (U.S. National Library of Medicine, Bethesda, MD, USA), Web of Science (Clarivate Analytics, Philadelphia, PA, USA), and IPCS/INCHEM (International Programme on Chemical Safety/Chemical Safety Information from Intergovernmental Organizations, WHO, Geneva, Switzerland). Reference lists of all articles were hand-searched for relevant studies not included in the original search results. The literature sources (including abstracts) were evaluated using Mendeley (Mendeley Inc., New York, NY, USA). By manual screening, relevant articles were identified and ordered in full-text. No unpublished study was identified. 

Additional data on the occurrence of furfuryl alcohol was also obtained from in-house analysis of 30 coffee (roasted coffee as beans, powder, or pods), 15 bread, 20 wine, and 50 alcoholic spirit samples (whiskey, brandy, and rum) submitted to our laboratory in the context of official control using nuclear magnetic resonance spectroscopy (NMR) [27]. For this, spectra previously acquired for other purposes were re-quantified for furfuryl alcohol. The coffee samples were analysed according to Monakhova et al. [28]. Quantification was conducted using the integral of the CH group at the C5 resonance of furfuryl alcohol (δ 7.47–7.35 ppm) in relation to the internal standard 1,2,4,5-tetrachloro-3-nitrobenzene (δ 7.75–7.72 ppm). Quantification was conducted using TopSpin 3.2 (BrukerBioSpin GmbH, Rheinstetten, Germany) and Mestrenova V. 11.0.2 (Mestrelab Research, Santiago de Compostela, Spain) [29]. For evaluation of spirits, the NMR method of Monakhova et al. [27] was applied. The NMR methods achieved a limit of detection (LOD) of 3.2 mg/L and limit of quantification (LOQ) of 8.6 mg/L. The results of NMR must be interpreted as semi-quantitative, because only one single non-overlapped signal of furfuryl alcohol was available for quantification. Identity was confirmed by spiking with pure furfuryl alcohol to authentic samples, but co-occurrence of compounds with a similar chemical shift cannot be completely excluded. The statistical parameters of mean, median, and percentiles (90th, 95th, 97.5th, and 99th) were used to describe the occurrence data. Similar NMR analysis of β-myrcene (e.g., in hops) was not possible due to considerable matrix interferences of all relevant signals. The concentration of β-myrcene in compounded products such as beer was below the detection limit of NMR.

## 3. Results

**Figure 1:**
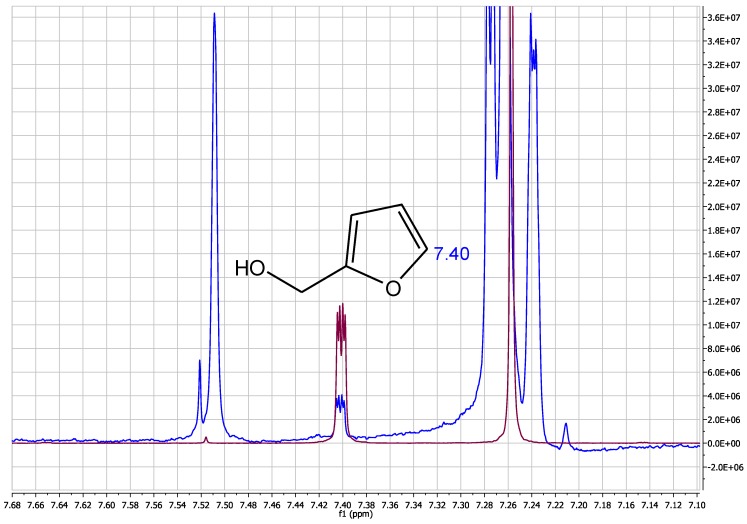
NMR spectra of an authentic coffee sample (**blue line**) containing 408 mg/kg of furfuryl alcohol compared to the reference standard (**red line**).

This study summarizes the occurrence of furfuryl alcohol and β-myrcene in various foods and beverages. Limited studies on β-myrcene (7) were observed compared to 19 studies for furfuryl alcohol. Meta-analysis was not possible due to the sparsity of studies for each type of food and beverage. The occurrence of furfuryl alcohol was recorded in many foods and beverages that had been subjected to thermal processing. The literature studies summarized in Table 1 were extended by inclusion of original results from our own analyses on furfuryl alcohol in 30 coffee, 15 bread, 20 wine, and 50 aged alcoholic spirit samples. From these, only coffee samples were positive (average furfuryl alcohol content of 251 mg/kg), while all other samples were below the detection limit of the method. A typical spectrum of a coffee sample is shown in Figure 1.

Out of the seven studies on β-myrcene, four were in hops and related products, while two were in beer, and the final reference reported about general use levels in various foods/beverages. Chewing gum, gelatin, beer, and hops were suggested as products with high concentration of β-myrcene. The studies are summarized in Table 2.

## 4. Discussion

### 4.1. Occurrence of Furfuryl Alcohol

The concentration of furfuryl alcohol was highest in coffee (beans 564 mg/kg and 267 mg/kg in instant coffee powder). Our new data on coffee with an average of 251 mg/kg and a maximum of 408 mg/kg corresponds well to the previous data. Green coffee is free of furfuryl alcohol (confirmed in eight samples with non-detectable levels), so the occurrence of furfuryl alcohol in coffee has been confirmed as being attributable to the roasting process [5]. This observation parallels the high content of furan found in coffee compared to other foods [24]. Other thermally processed foods, such as bread (187 mg/kg), baked goods (110 mg/kg), ice cream/ices (88 mg/kg), and fried fish (about 10 mg/kg) were also found to contain detectable amounts of furfuryl alcohol. Among beverages, higher concentrations of furfuryl alcohol arising from aging in oak barrels [30] were found in spirits (10 mg/L) than in wine (1.5–3.4 mg/L). However, the content was lower compared to bread, baked goods, fish, and coffee. Relatively lower concentrations (less than 1 mg/kg) were observed in palm sugar, chips, popcorns, sweet potatoes, and vinegar. The variation in the concentration of furfuryl alcohol in the foods/beverages may be related to the type of raw materials and processing conditions used. The Joint FAO/WHO Expert Committee on Food Additives (JECFA) set a group acceptable daily intake (ADI) of 0–0.5 mg/kg body weight for furfuryl alcohol, and suggested the compound as being of no safety concern at current levels of intake when used as a flavouring agent [48]. Despite the concentrations reported here being low for a majority of individual foods and beverages, a cumulative amount of furfuryl alcohol may be ingested from consuming a combination of different foods and beverages. According to the National Toxicology Program (NTP) report [49], exposure of male mice to 32 ppm (equivalent to 60 mg/kg bw/day [50]) of furfuryl alcohol was found to induce tumours of renal tubular epithelium. The postulated mechanism of carcinogenicity of furfuryl alcohol is through activation by sulfotransferases resulting in the formation of a 2-methylfuranyl-DNA adduct [50,51]. According to estimation from the typical intake levels of the food items listed in Table 1, concentrations of toxicological concern are probably not reached. However, food legislation demands to reduce food contaminants as low as reasonably achievable (ALARA principle). More data are clearly necessary to provide exposure estimations and risk assessment for this compound.

### 4.2. Occurrence of β-Myrcene

A majority of the studies on β-myrcene are qualitative, and the few quantitative data were focusing on hops and beers, despite the widespread occurrence of myrcene in many plants that are used in foods and beverages. Hop oil and chewing gum were found to contain the highest content of β-myrcene compared to other products. The low concentration of β-myrcene in beers is plausible, since there is a very variable extraction of β-myrcene from hops to beer postulated to be in the range of 0.5%–5.6% from cones and 8.4%–25.8% from pellets [52], and hops contain other volatile components such linanool, humulene, and α-terpineol in higher proportions than β-myrcene. Additionally, β-myrcene may be destroyed during the heating processes, and thus a low level is expected in the final beer. The NTP report links β-myrcene with neoplasms of the kidney in male rats and liver cancer in male mice [53]. The daily per capita intake (eaters only) for β-myrcene was estimated as being 164 µg corresponding to 3 µg/kg bw [54]. 

## 5. Conclusions 

Consistent with the relatively high amounts of furfuryl alcohol (above 10 mg/kg) observed in coffee, baked goods, bread, fish, and some spirit drinks, monitoring these items for furfuryl alcohol is advisable for comprehensive estimation of exposures and the risk of these foods, while more research on the occurrence of β-myrcene in foods and beverages in general is required for meaningful risk assessment. 

## Figures and Tables

**Table 1:** Furfuryl alcohol content in various foods and beverages.

Category [Reference]	N	Furfuryl Alcohol Concentration							Units ^a^
		Mean	Median	P90	P95	P97.5	P99	Maximum	
Roasted coffee/This study	30	251	243	342	392	402	406	408	mg/kg
Bread/This study	15	<LOD ^b^	-	-	-	-	-	-	mg/kg
Wine/This study	20	<LOD ^b^	-	-	-	-	-	-	mg/L
Spirits/This study	50	<LOD ^b^	-	-	-	-	-	-	mg/L
Sweet potatoes [4]	1	0.014	-	-	-	-	-	-	mg/kg
Wine [7]	8	3.4	2.9	7.3	8.5	9.0	9.4	9.6	mg/L
Baked goods [9] ^d^	-	110	-	-	-	-	-	-	ppm
Spirits [9] ^d^	-	10	-	-	-	-	-	-	ppm
Candy [9] ^d^	-	59	-	-	-	-	-	-	ppm
Ice cream/ices [9] ^d^	-	88	-	-	-	-	-	-	ppm
Beverages [9] ^d^	-	19	-	-	-	-	-	-	ppm
Honey [14]	1	1.6	-	-	-	-	-	-	mg/kg
Popcorns [15]	6	0.064	0.067	0.081	0.081	0.082	0.082	0.082	mg/kg
Fried fish [16]	1	10.5	-	-	-	-	-	-	mg/kg
Breaded fish products [17]	4	10.3	8.8	16	18	18	19	19	mg/kg
Wine [30]	6	1.51	0.89	1.57	1.60	1.62	1.63	1.64	mg/L
Vinegar [31] ^c^	27	0.35	0.28	0.58	0.59	0.59	0.59	0.59	mg/L
Vinegar [32] ^c^	9	0.34	0.28	0.58	0.59	0.59	0.59	0.59	mg/L
Coffee [33]	7	49	49	64	67	68	69	70	mg/kg
Instant coffee [34]	1	267	-	-	-	-	-	-	mg/kg
Roasted coffee [34]	1	564	-	-	-	-	-	-	mg/kg
Pineapple juice [34]	1	8.3	-	-	-	-	-	-	mg/L
Rice cakes [35]	2	2, 2.3	-	-	-	-	-	2.3	mg/kg
Bread [36]	1	187	-	-	-	-	-	-	mg/kg
Toasted almonds [37]	3	6.4	6.0	8.3	8.6	8.7	8.8	8.9	mg/kg
Non-fat dried milk [38]	1	15	-	-	-	-	-	-	mg/kg
Corn tortilla chips [39]	1	0.54	-	-	-	-	-	-	mg/kg
Cocoa powder [40]	1	0.02	-	-	-	-	-	-	mg/kg
Palm sugar [41]	1	0.14, 0.52	-	-	-	-	-	-	mg/kg

^a^ The ambiguous unit ppm was interpreted as mg/L for liquids/beverages and as mg/kg for solid foods. ^b^ All samples evaluated (spirits types whiskey, rum, brandy as well as various wines and breads) were below the limit of detection (LOD; 3.2 mg/L). ^c^ Studies from the same research group with probably overlapping data. ^d^ Number of samples not provided. The data are suggested as being “usual concentrations” found in these food/beverage types.

**Table 2:** β-Myrcene content in various matrices.

Matrix [Reference]	N	Concentration							Units ^a^
		Mean	Median	P90	P95	P97.5	P99	Maximum	
Hops oil [18]	4	479	424	776	852	890	912	927	mg/L
Hops [42]	12	5489	4804	8580	9450	9972	10,285	10,494	mg/kg
Hops [43]	8	15	14	28	29	29	29	29	µg/L
Hops [44]	12	1082	705	2369	2795	3043	3191	3290	mg/kg
Pilsner beer [45]	2	46, 79	-	-	-	-	-	79	µg/L
Beer [46]	2	0.5, 0.6	-	-	-	-	-	0.6	µg/L
Alcoholic beverages [47] ^b^	-	1.1	-	-	-	-	-	-	mg/L
Baked goods [47] ^b^	-	10	-	-	-	-	-	-	mg/kg
Chewing gum [47] ^b^	-	116	-	-	-	-	-	-	mg/kg
Condiment [47] ^b^	-	5	-	-	-	-	-	-	mg/kg
Frozen dairy [47] ^b^	-	12	-	-	-	-	-	-	mg/kg
Gelatin, pudding [47] ^b^	-	20	-	-	-	-	-	-	mg/kg
Meat products [47] ^b^	-	5	-	-	-	-	-	-	mg/kg
Non-alcoholic beverages [47] ^b^	-	8	-	-	-	-	-	-	mg/L
Soft candy [47] ^b^	-	6	-	-	-	-	-	-	mg/kg

^a^ The ambiguous unit ppm was interpreted as mg/L for liquids/beverages and as mg/kg for solid food. ^b^ Number of samples not provided. The data are suggested as being “usual concentrations” found in these food/beverage types.

## References

[1] Monakhova Y.B., Lachenmeier D.W. (2012). The Margin of Exposure of 5-Hydroxymethylfurfural (HMF) in alcoholic beverages. Environ. Health Toxicol..

[2] Martins S.I., Jongen W.M., Boekel M.A. (2001). A review of Maillard reaction in food and implications to kinetic modelling. Trends Food Sci. Technol..

[3] Vanderhaegen B., Delvaux F., Daenen L., Verachtert H., Delvaux F.R. (2007). Aging characteristics of different beer types. Food Chem..

[4] Wang Y., Kays S.J. (2000). Contribution of volatile compounds to the characteristic aroma of baked “Jewel” sweetpotatoes. J. Amer. Soc. Hort. Sci..

[5] Swasti Y.R., Murkovic M. (2012). Characterization of the polymerization of furfuryl alcohol during roasting of coffee. Food Funct..

[6] Pérez-Prieto L.J., López-Roca J.M., Martínez-Cutillas A., Pardo-Mínguez F., Gómez-Plaza E. (2003). Extraction and formation dynamic of oak-related volatile compounds from different volume barrels to wine and their behavior during bottle storage. J. Agric. Food Chem..

[7] Spillman P.J., Pollnitz A.P., Liacopoulos D., Pardon K.H., Sefton M.A. (1998). Formation and degradation of furfuryl alcohol, 5-Methylfurfuryl alcohol, vanillyl alcohol, and their ethyl ethers in barrel-aged wines. J. Agric. Food Chem..

[8] Mochizuki N., Kitabatake K. (1997). Analysis of 1-(2-furyl)propane-1,2-diol, a furfural metabolite in beer. J. Ferment. Bioeng..

[9] National Research Council (U.S.) (1965). Chemicals used in Food Processing.

[10] Paumgartten F.J., De-Carvalho R.R., Souza C.A., Madi K., Chahoud I. (1998). Study of the effects of beta-myrcene on rat fertility and general reproductive performance. Braz. J. Med. Biol. Res..

[11] Mohamed Hanaa A.R., Sallam Y.I., El-Leithy A.S., Aly S.E. (2012). Lemongrass (Cymbopogon citratus) essential oil as affected by drying methods. Ann. Agric. Sci..

[12] Lachenmeier K., Musshoff F., Madea B., Lachenmeier D.W. (2006). Application of experimental design to optimise solid-phase microextraction of orange juice flavour. Electron. J. Environ. Agric. Food Chem..

[13] Behr A., Johnen L. (2009). Myrcene as a natural base chemical in sustainable chemistry: A critical review. ChemSusChem.

[14] Vázquez L., Verdú A., Miquel A., Burló F., Carbonell-Barrachina A.A. (2007). Changes in physico-chemical properties, hydroxymethylfurfural and volatile compounds during concentration of honey and sugars in Alicante and Jijona turron. Eur. Food Res. Technol..

[15] Park D., Maga J.A. (2006). Identification of key volatiles responsible for odour quality differences in popped popcorn of selected hybrids. Food Chem..

[16] Pérez-Palacios T., Petisca C., Melo A., Ferreira I.M. (2012). Quantification of furanic compounds in coated deep-fried products simulating normal preparation and consumption: Optimisation of HS-SPME analytical conditions by response surface methodology. Food Chem..

[17] Pérez-Palacios T., Petisca C., Henriques R., Ferreira I.M. (2013). Impact of cooking and handling conditions on furanic compounds in breaded fish products. Food Chem. Toxicol..

[18] Van Opstaele F., De Causmaecker B., Aerts G., De Cooman L. (2012). Characterization of novel varietal floral hop aromas by headspace solid phase microextraction and gas chromatography-mass spectrometry/olfactometry. J. Agric. Food Chem..

[19] Gonçalves J., Figueira J., Rodrigues F., Câmara J.S. (2012). Headspace solid-phase microextraction combined with mass spectrometry as a powerful analytical tool for profiling the terpenoid metabolomic pattern of hop-essential oil derived from Saaz variety. J. Sep. Sci..

[20] Roberts M.T., Dufour J.P., Lewis A.C. (2004). Application of comprehensive multidimensional gas chromatography combined with time-of-flight mass spectrometry (GC x GC-TOFMS) for high resolution analysis of hop essential oil. J. Sep. Sci..

[21] Kishimoto T., Wanikawa A., Kono K., Shibata K. (2006). Comparison of the odor-active compounds in unhopped beer and beers hopped with different hop varieties. J. Agric. Food Chem..

[22] Lachenmeier D.W., Przybylski M.C., Rehm J. (2012). Comparative risk assessment of carcinogens in alcoholic beverages using the margin of exposure approach. Int. J. Cancer.

[23] Lachenmeier D.W. (2009). Carcinogens in Food: Opportunities and Challenges for Regulatory Toxicology. Open Toxicol. J..

[24] Waizenegger J., Winkler G., Kuballa T., Ruge W., Kersting M., Alexy U., Lachenmeier D.W. (2012). Analysis and risk assessment of furan in coffee products targeted to adolescents. Food Addit. Contam..

[25] European Food Safety Authority (2008). Polycyclic Aromatic Hydrocarbons in Food. EFSA J..

[26] Singh L., Varshney J.G., Agarwal T. (2016). Polycyclic aromatic hydrocarbons’ formation and occurrence in processed food. Food Chem..

[27] Monakhova Y.B., Schäfer H., Humpfer E., Spraul M., Kuballa T., Lachenmeier D.W. (2011). Application of automated eightfold suppression of water and ethanol signals in 1H NMR to provide sensitivity for analyzing alcoholic beverages. Magn. Reson. Chem..

[28] Monakhova Y.B., Ruge W., Kuballa T., Ilse M., Winkelmann O., Diehl B., Thomas F., Lachenmeier D.W. (2015). Rapid approach to identify the presence of Arabica and Robusta species in coffee using 1H NMR spectroscopy. Food Chem..

[29] Bernstein M.A., Sýkora S., Peng C., Barba A., Cobas C. (2013). Optimization and automation of quantitative NMR data extraction. Anal. Chem..

[30] Carrillo J.D., Garrido-López Á., Tena M.T. (2006). Determination of volatile oak compounds in wine by headspace solid-phase microextraction and gas chromatography-mass spectrometry. J. Chromatogr. A.

[31] Morales M.L., Benitez B., Troncoso A.M. (2004). Accelerated aging of wine vinegars with oak chips: Evaluation of wood flavour compounds. Food Chem..

[32] Tesfaye W., Morales M.L., Benítez B., García-Parrilla M.C., Troncoso A.M. (2004). Evolution of wine vinegar composition during accelerated aging with oak chips. Anal. Chim. Acta.

[33] Petisca C.I. (2013). Furanic Compounds in Food Products: Assessment and Mitigation Strategies. Ph.D. Thesis.

[34] Golubkova T. (2011). Bildung von Potentiell Toxischen Furanderivaten in Lebensmitteln. Masters Thesis.

[35] Moon J.K., Shibamoto T. (2009). Role of roasting conditions in the profile of volatile flavor chemicals formed from coffee beans. J. Agric. Food Chem..

[36] Jensen S., Ostdal H., Skibsted L.H., Thybo A.K. (2011). Antioxidants and shelf life of whole wheat bread. J. Cereal Sci..

[37] Vázquez-Araújo L., Enguix L., Verdú A., García-García E., Carbonell-Barrachina A.A. (2008). Investigation of aromatic compounds in toasted almonds used for the manufacture of turrón. Eur. Food Res. Technol..

[38] Karagül-Yüceer Y., Cadwallader K.R., Drake M.A. (2002). Volatile flavor components of stored nonfat dry milk. J. Agric. Food Chem..

[39] Buttery R.G., Ling L.C. (1998). Additional Studies on Flavor Components of Corn Tortilla Chips. J. Agric. Food Chem..

[40] Bonvehí J.S. (2005). Investigation of aromatic compounds in roasted cocoa powder. Eur. Food Res. Technol..

[41] Ho C.W., Aida W.M.W., Maskat M.Y., Osman H. (2007). Changes in volatile compounds of palm sap (Arenga pinnata) during the heating process for production of palm sugar. Food Chem..

[42] Aberl A., Coelhan M. (2012). Determination of volatile compounds in different hop varieties by headspace-trap GC/MS—In comparison with conventional hop essential oil analysis. J. Agric. Food Chem..

[43] Mitter W., Cocuzza S. Dry Hopping—A Study of Various Parameters Consequences of the Applied Dosing Method. http://hopsteiner.com/wp-content/uploads/2016/03/3_Dry-Hopping-A-Study-of-Various-Parameters.pdf.

[44] Peacock V.E., Deinzer M.L. (1979). Chemistry of hop aroma in beer. J. Am. Soc. Brew. Chem.

[45] Schmidt C., Biendl M. (2016). Headspace Trap GC-MS analysis of hop aroma compounds in beer. BrewingScience.

[46] Mikyška A., Olšovská J. Czech Research and Development in the Field of Brewing Raw Materials. http://www.hmelj-giz.si/ihgc/doc/LdVBS-RIBM_raw_materials_research.pdf.

[47] Burdock G.A. (2004). Fenaroli’s Handbook of Flavor Ingredients.

[48] World Health Organization (WHO) (2001). Evaluation of Certain Food Additives and Contaminants.

[49] National Toxicology Program (NTP) (1999). Technical Report on the Toxicology and Carcinogenesis Studies of Furfuryl Alcohol (CAS NO. 98-00-0) in F344/N Rats and B6C3F1 Mice (Inhalational Studies). NTP TR 482. NIH Publication No. 99-3972.

[50] Sachse B., Meinl W., Sommer Y., Glatt H., Seidel A., Monien B.H. (2016). Bioactivation of food genotoxicants 5-hydroxymethylfurfural and furfuryl alcohol by sulfotransferases from human, mouse and rat: A comparative study. Arch. Toxicol..

[51] Sachse B., Meinl W., Glatt H., Monien B.H. (2014). The effect of knockout of sulfotransferases 1a1 and 1d1 and of transgenic human sulfotransferases 1A1/1A2 on the formation of DNA adducts from furfuryl alcohol in mouse models. Carcinogenesis.

[52] Wolfe P.H. (2012). A Study of Factors Affecting the Extraction of Flavor When Dry Hopping Beer. Masters Thesis.

[53] National Toxicology Program (NTP) (2011). Technical Report on the Toxicology and Carcinogenesis Studies of β-myrcene (CAS NO. 123-35-3) in F344/N Rats and B6C3F1 Mice. NTP TR 557. NIH Publication No. 09-5898.

[54] Adams T.B., Gavin C.L., McGowen M.M., Waddell W.J., Cohen S.M., Feron V.J., Marnett L.J., Munro I.C., Portoghese P.S., Rietjens I.M.C.M., Smith R.L. (2011). The FEMA GRAS assessment of aliphatic and aromatic terpene hydrocarbons used as flavor ingredients. Food Chem. Toxicol..

